# Co-administration of vitamin D and N-acetylcysteine to modulate immunosenescence in older adults with vitamin D deficiency: a randomized clinical trial

**DOI:** 10.3389/fimmu.2025.1570441

**Published:** 2025-05-12

**Authors:** Samira Rastgoo, Katayoun Pourvali, Seyed Ahmad Raeissadat, Ghazaleh Eslamian, Hamid Zand

**Affiliations:** ^1^ Department of Cellular and Molecular Nutrition, Faculty of Nutrition and Food Technology, National Nutrition and Food Technology Research Institute, Shahid Beheshti University of Medical Sciences, Tehran, Iran; ^2^ Physical Medicine and Rehabilitation Department, Shahid Modarres Hospital, Shahid Beheshti University of Medical Sciences, Tehran, Iran

**Keywords:** immune function, geroscience, cellular senescence, nutrition, aging

## Abstract

**Background:**

Immunosenescence is an important factor in the impaired immune response in older adults and plays a significant role in the development of biological aging. Targeting immunosenescence could present a novel pharmacological approach to mitigating aging and age-related diseases. We aimed to investigate the effect of N-acetylcysteine (NAC) and vitamin D (Vit-D) on the senescence of peripheral blood mononuclear cells (PBMCs).

**Method:**

This randomized clinical trial was conducted on older adults with Vit-D deficiency. Eligible participants were randomly assigned to one of four groups to receive either (A) 1000 IU of Vit-D daily (D1) (B), 1000 IU of Vit-D plus 600 mg of NAC daily (D1N) (C), 5000 IU of Vit-D daily (D5), or (D) 5000 IU of Vit-D plus 600 mg of NAC daily (D5N) for 8 weeks. Senescence-associated beta-galactosidase (SA-β-gal) staining, expression of senescence-related genes, and serum inflammatory factors were measured at baseline and after 8 weeks.

**Results:**

After the intervention, supplementation with D5N and D5 significantly downregulated *p16*, interleukin-6 (*IL-6*), and tumor necrosis factor-α (*TNF-α*) expression and decreased SA-β-gal activity compared to the D1 group. Additionally, co-administration of NAC with 1000 IU of Vit-D significantly downregulated *p16* transcripts in PBMCs compared to Vit-D 1000 IU alone. No significant differences were observed between the groups in serum IL-6, C-reactive protein (CRP), or the neutrophil-to-lymphocyte ratio (NLR) after the intervention.

**Conclusions:**

The loading dose of Vit-D significantly attenuates senescence in PBMCs of older adults. However, co-administration of NAC with both the standard and loading doses of Vit-D further enhances these beneficial effects.

**Clinical trial registration:**

https://irct.behdasht.gov.ir, identifier IRCT20230508058120N1.

## Introduction

The aging population is growing in many countries (1). As people age, they become more vulnerable to age-related diseases such as cardiovascular diseases, strokes, diabetes, neurodegenerative disorders, and cancers (2). Understanding the mechanisms that contribute to aging in the body can help slow the aging process, increase life expectancy, and reduce the burden of age-related diseases on national health systems. Recent evidence strongly suggests that the accumulation of senescent cells drives aging at the organismal level. Senescence is a cellular state in which cells lose their proliferative capacity and release specific humoral factors, including inflammatory cytokines, growth factors, matrix metalloproteinases, and factors involved in tissue regeneration and fibrosis. This secretory process is known as the senescence-associated secretory phenotype (SASP). The specific components of SASP depend on the cell type and its context (3, 4). Although post-mitotic differentiated cells typically do not proliferate, they can still undergo senescence, exhibiting only the SASP phenotype. Various stressors can induce senescence in cells, including DNA-damaging factors, oncogene activation, oxidative stress, and telomere shortening (5, 6). Senescence is a physiological phenomenon that prevents the proliferation and persistence of damaged cells. However, consistent with the antagonistic theory of aging, mechanisms like senescence that promote fitness in early life may have detrimental effects later in life (7). The accumulation of senescent cells in older organisms contributes to the etiology of aging and related diseases (8).

Senescent cells are cleared through the induction of apoptosis or an immune response (3, 9). The immune system plays a critical role in the removal of senescent cells throughout the body. In the context of senescence, cells influence the innate immune response by releasing inflammatory molecules as part of the SASP. This process typically promotes the growth and activation of M1 macrophages while inhibiting M2 macrophages. In response to the heightened inflammation, natural killer (NK) cells are recruited to areas where senescent cells are present, as these cells display NK-stimulating molecules on their surface. The interaction between these NK ligands on senescent cells and the corresponding NK receptors on NK cells leads to the elimination of senescent cells (10). Any dysfunction or weakening of the immune response impairs the removal of senescent cells (9). With aging, accumulated challenges—such as pathogens, tissue damage, and cellular stress—trigger inflammatory responses that, if not properly regulated by anti-inflammatory processes, can drive the onset and progression of inflammaging, along with an increase in senescent cells. Inflammaging occurs alongside and as a consequence of age-related declines in immune function, a process known as immunosenescence (11). In addition to systemic aging, immunosenescence may also result from the transmission of senescence, driven by the accumulation of senescent cells in other tissues through the SASP. Older individuals are often more susceptible to infectious diseases, experience delayed wound healing, and exhibit impaired vaccine-induced immunity. Most of these complications stem from immunosenescence (2, 12). Immunosenescence is the main factor for the weakness of the immune response when faced with the accumulation of senescence in other tissues. Therefore, immunosenescence is a significant factor in the progression of biological aging. Preventing or reversing immunosenescence could be a novel pharmacological target to slow aging and reduce age-related diseases (13, 14). We recently demonstrated that the accumulation of senescent cells in the adipocytes of obese individuals decreases after 4 weeks of oral consumption of 600 milligrams of N-acetylcysteine (NAC), an over-the-counter (OTC) drug (15). NAC is used as a mucolytic agent and to treat liver poisoning caused by paracetamol overdose. Recent studies suggest that NAC is a potent antioxidant—likely due to its role in glutathione synthesis—and exhibits anti-inflammatory properties (16). Immunosenescence is exacerbated by deficiencies in certain nutrients, such as vitamin D (Vit-D). Vit-D is an immune-modulating hormone that may serve as a potential tool to mitigate immunosenescence (17).

In this study, we aim to investigate the effects of NAC and Vit-D in a clinical trial on the senescence of human peripheral blood mononuclear cells (PBMCs). We will use a simple method—the senescence-associated beta-galactosidase (SA-β-gal) staining assay—along with the analysis of senescence-related gene expression to assess senescence in the PBMCs of older individuals. More than 60% of the PBMC population consists of T cells, while the remaining cells include NK cells, monocytes, and B cells (18). Since T cells are more susceptible to senescence due to their high proliferative capacity, we considered PBMC senescence a biomarker of immunosenescence.

## Methods

### Trial design and participant

The study was a double-blind, randomized controlled trial. Participants were recruited between October 2023 and June 2024 from community centers and the Physical Medicine and Rehabilitation Clinic of Shahid Modarres Hospital in Tehran, Iran. Eligible participants provided written informed consent after receiving a comprehensive explanation of the study procedures, including the potential risks and benefits of the intervention. The study protocol was approved by the Shahid Beheshti University Ethics Committee and registered in the Iranian Registry of Clinical Trials under registration number IRCT20230508058120N1.

Sample size calculations were performed to compare the Vit-D 5000 IU group with the control (Vit-D 1000 IU) and the NAC + Vit-D 1000 IU group with the control. The final sample size was determined based on the larger of the two calculations, with an additional 10% to account for potential participant loss. The minimum estimated sample size for each group was 22, accounting for potential sample loss. This sample size was sufficient to evaluate the main effects with a power (1 − β) of 80% and α = 0.05 in a two-arm parallel study, allowing detection of a 5.5 pg/ml difference in serum interleukin-6 (IL-6) levels based on previous studies (19, 20).

To detect Vit-D deficiency, serum samples were collected from all participants before randomization. The criterion for diagnosing Vit-D deficiency was a serum 25-hydroxy Vit-D [25(OH)D] level below 30 ng/ml (75 nmol/l) (21, 22).

The inclusion criteria were Vit-D deficiency, age above 65 years, and a body mass index (BMI) between 25 and 35 kg/m², which is considered the optimal range for older adults based on the study by Kıskaç et al. (23).

Exclusion criteria included chronic or acute inflammatory and infectious diseases, diabetes, thyroid disorders, Alzheimer’s disease, dementia, and the use of medications that affect Vit-D metabolism, such as anticonvulsants, antituberculosis drugs, and glucocorticoids. Other exclusion factors were hypercalcemia, electrolyte imbalances, arrhythmia, renal or hepatobiliary dysfunction, asthma, gastric hemorrhage, fluid overload, and the use of hydrochlorothiazide or magnesium oxide. Additionally, participants were excluded if they were enrolled in other research that could interfere with participation or data interpretation, had used antioxidants, NAC, or Vit-D supplements in the past 3 months, or consumed alcohol or smoked.

### Randomization and interventions

**Figure 1:**
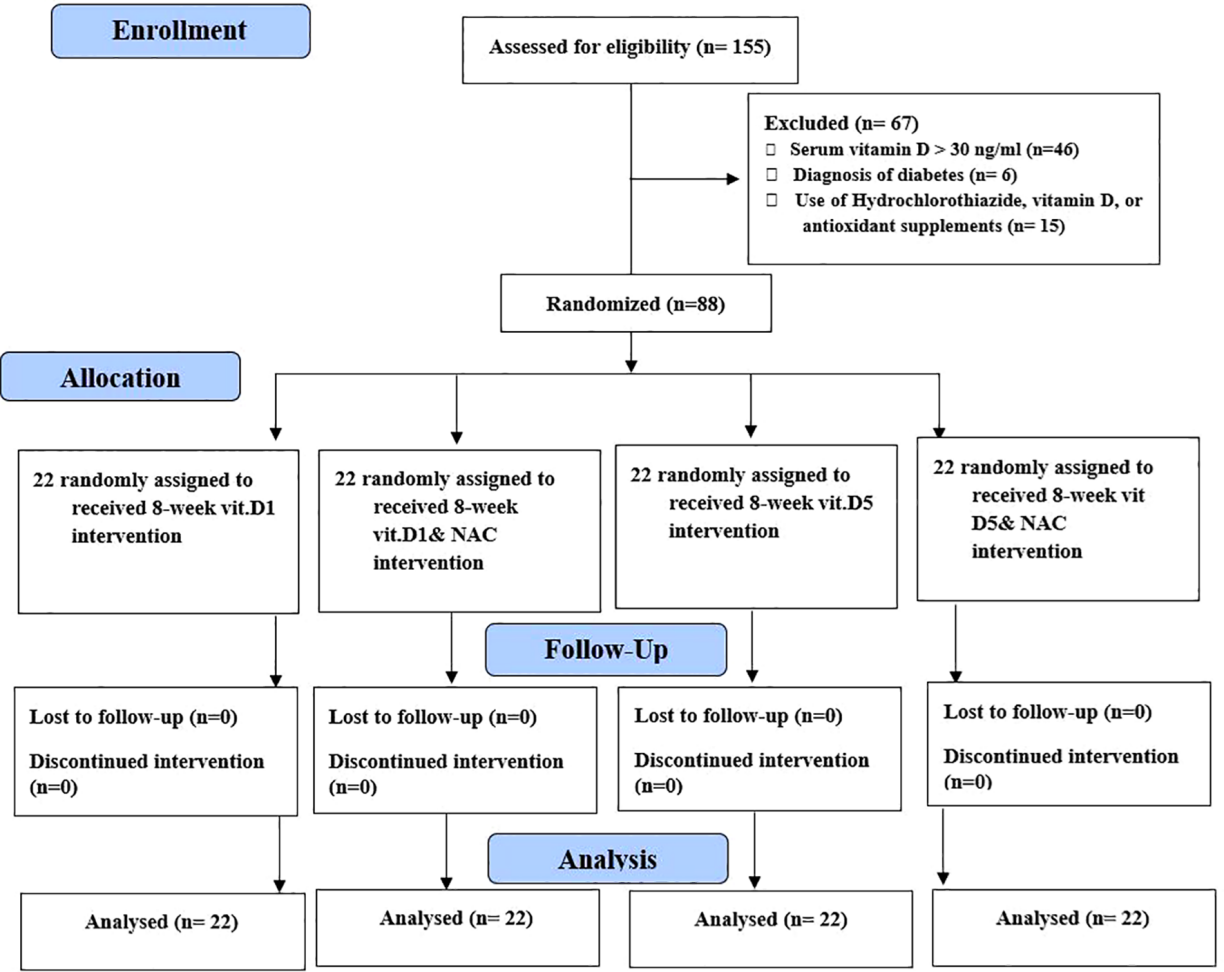
Flowchart of participants. NAC, N-acetylcysteine; D1, vitamin D1000 IU; D5, vitamin D5000 IU.

Details about recruitment, randomization, and follow-up are provided in **Figure 1**. Randomization was stratified by gender (female and male) and serum 25(OH)D levels (< 10, 10–19, and 20–30 ng/ml). The randomization list was generated using the online software available at www.sealedenvelope.com.

The following reasons justify the selection of supplemental doses for NAC and Vit. D:

A systematic review and meta-analysis reported that NAC supplementation doses used to reduce inflammatory biomarkers ranged from 600 to 1,800 mg, with intervention durations ranging from 5 days to 12 months. The most significant effect was observed at doses below 1,000 mg, with minimum intervention duration of 4 weeks (24).

Similarly, numerous studies and guidelines recommend a daily dose of 4000 IU of Vit-D (cholecalciferol) for 8–12 weeks as the preferred supplementation strategy for treating Vit-D deficiency. However, to ensure that serum 25(OH)D levels exceeded 30 ng/ml in Vit-D–deficient older adults, a loading dose of 5000 IU/day was administered (21, 25).

Additionally, following the recommendations of the Endocrine Society and the International Osteoporosis Foundation, two study groups received a maintenance dose of 1000 IU/day of Vit-D. These organizations suggest that a daily dose of 600–800 IU may help older adults achieve serum 25(OH)D levels of 30 ng/ml (26).

Participants were assigned to one of four groups for an 8-week intervention: a control group receiving 1000 IU/day of Vit-D (D1), 1000 IU of Vit-D plus 600 mg of NAC (D1N), 5000 IU of Vit-D (D5), or 5000 IU of Vit-D plus 600 mg of NAC (D5N). This time frame was chosen as the minimum duration required to increase serum Vit-D levels in individuals receiving therapeutic doses (25, 27).

The supplements were identical in shape and packaging and labeled A, B, C, and D by Pharmachemie Pharmaceutical Company (Tehran, Iran). Group assignment codes were concealed in sealed envelopes and opened at enrollment by a third party blinded to all baseline assessments. Neither participants nor investigators knew the group assignments until the study concluded.

### Treatment and follow-up

A physician and a nutritionist assessed participants at baseline and again in the eighth week after the intervention. Evaluations included blood sample collection, protocol adherence assessment, and monitoring for supplement side effects. Participants received weekly follow-up phone calls to encourage adherence, record any side effects, and address study-related questions. Supplements were distributed every 4 weeks, and compliance was assessed by counting the remaining supplements.

### Procedures

At the screening visit, baseline characteristics were collected through questionnaires covering demographic data, medical history, smoking status, current medications, and supplement use (**Supplementary S1**).

### Anthropometric measurements

Before and after the study, weight, height, and waist circumference (WC) were measured for all participants. BMI was calculated as weight divided by height squared (kg/m²). To minimize measurement errors, all assessments were conducted by a single examiner.

### Dietary intake assessments and physical activity

To assess food intake, all participants completed a 3-day food record, including one weekend day and two weekdays, at the beginning of the study, in the fourth week, and at the end of the study. The recorded dietary data were analyzed using Nutritionist 4 software (First Databank, San Bruno, CA, USA), which was modified for Iranian foods by an expert dietitian.

Physical activity was assessed using the Rapid Assessment of Physical Activity (RAPA) questionnaire, a valid, easy-to-administer, and interpretable tool for evaluating physical activity levels in adults over 50 (28).

### PBMC isolation and SA-β-gal staining

PBMCs were isolated from whole blood using the Ficoll density gradient centrifugation method. The extracted PBMCs were divided into two portions: 20% was used fresh for the SA-β-gal staining assay, while the remaining 80% was stored at −80°C for mRNA extraction.

SA-β-galactosidase activity was assessed using a cytochemical staining protocol (**Supplementary S2**) (29), standardized as follows:

1. Cell pellets were suspended in 50 μl of fixation buffer at 4°C for 15 min.
2. The samples were centrifuged.
3. After the third PBS wash, the supernatant was discarded, and the cell pellet was resuspended in 50–100 μl of staining solution, depending on the cell count.
4. The solution was incubated overnight at 37°C.
5. Following incubation, the samples were centrifuged and washed three times with PBS.
6. Finally, slides were prepared from the PBS-suspended cells, and images were captured using a light microscope.

The percentage of green pixels relative to the total pixels in the calibrated image was calculated using ImageJ software (National Institutes of Health, USA). This ratio represents the green area ratio, which was used to quantify SA-β-gal activity.

### RNA extraction, cDNA synthesis, and quantitative real-time polymerase chain reaction

RNA extraction and complementary DNA (cDNA) synthesis were performed according to the manufacturer’s instructions for the respective kits. Total RNA was extracted from PBMCs using RNX Plus solution (Cinaclone, Tehran Iran). For cDNA synthesis (SMOBIO kit, Taiwan), 1 μg of total RNA was used. Polymerase chain reaction (PCR) was conducted to amplify *p16, p21, IL6*, tumor necrosis factor-α (*TNF-α*), and glyceraldehyde-3-phosphate dehydrogenase (GAPDH) (used as an internal control). Reactions were performed in a final volume of 20 μl, containing:

- 10 μl ExcelTaq™ 2X real-time PCR master mix (SYBR; Rox, SMOBIO, Taiwan).
- 7 μl double-distilled water.
- 0.5 μl forward primer (10 pmol/µl).
- 0.5 μl reverse primer (10 pmol/µl).
- 2 μl cDNA.

The PCR protocol included an initial denaturation at 95°C for 15 min, followed by 40 cycles of amplification. Denaturation and annealing were carried out at 95°C for 25 s and 60°C for 25 s, respectively. The melt curve ranged from 60°C to 95°C (Applied Biosystems, StepOnePlus; Real-Time PCR, UK).

### Biochemical assays

Venous blood samples were collected from each participant after 8h–10h of fasting. Blood was drawn into heparinized tubes for plasma separation and ethylenediaminetetraacetic acid (EDTA) tubes for a complete blood count with a differential test. Samples were obtained at both the beginning and the end of the study. Platelet-free plasma was isolated by centrifugation and stored at −80°C for biochemical analysis.

Concentrations of 25(OH)D, the major circulating form of Vit-D in blood, were measured using a competitive enzyme-linked immunosorbent assay (ELISA) kit (DiaPlus Inc., Toronto, Ontario, Canada).

C-reactive protein (CRP) concentrations were measured in batches using commercial kits from Pars-Azmoon (Karaj, Iran) with an automated analyzer (Selectra Pro XL, Vital Scientific, Spankeren, The Netherlands).

Plasma IL-6 levels were assessed using an ELISA kit (LDN, GmBH, Germany).

The neutrophil-to-lymphocyte ratio (NLR) was calculated by dividing the absolute neutrophil count by the absolute lymphocyte count, both obtained from peripheral blood samples.

### Primary and secondary outcomes

In this study, the primary outcome measures were serum IL-6 levels, SA-β-gal staining, and gene expression levels of *p16*, *p21*, *IL6*, and *TNF-α*, all serving as markers of cellular senescence and inflammation. Secondary outcome measures included serum concentrations of 25(OH)D, CRP, and NLR.

### Statistical analysis

Statistical analysis was performed using SPSS version 20 (SPSS, Inc.). All hypothesis tests were two-tailed, and the statistical significance level was set at *p* < 0.05. The Kolmogorov–Smirnov test with a significance level of 5% and histograms and Q–Q plots were used to test continuous variables for the normality assumption. If the variables were skewed and nonnormally distributed, we used transformations like logarithm, square root, etc., to apply parametric tests. Qualitative data were reported as frequencies and percentages. Quantitative data were described using means ± SDs. The chi-square test assessed differences in categorical variables between groups. Independent-sample t-tests compared means between two groups, and one-way analysis of variance evaluated mean differences across the four groups at baseline. Post-intervention comparisons were made using analysis of covariance, adjusting for baseline values and age, with the Bonferroni *post hoc* test applied when significant main effects were observed.

The ΔΔCT method was used in real-time PCR analysis. ΔCT refers to the difference between the target gene’s threshold cycles (CT) and the internal control gene, GAPDH. The ΔΔCT value was computed by subtracting the mean ΔCT changes of genes in the intervention groups from the mean changes in the control group. Gene expression levels are then determined as fold changes, defined by 2^−ΔΔct^.

## Results

### Recruitment and follow-up

Among the 155 participants screened for this trial, 88 were included and randomly assigned to four groups. All participants [D1 (*n* = 22), D5 (*n* = 22), D1N (*n* = 22), and D5N (*n* = 22)] completed the study, resulting in a 100% participation rate among older adults.

According to follow-ups conducted during the study and supplement counts, compliance with the study protocol exceeded 90% in all groups. More details on recruitment, randomization, and follow-up are provided in **Figure 1**.

### Baseline characteristics

**Table 1** presents the baseline demographic characteristics, along with dietary and physical activity information. The mean age and BMI of the participants were 69.30 ± 4.14 years (range: 65–82 years) and 27.40 ± 1.77 kg/m² (range: 25–32 kg/m²), respectively. No significant differences were observed between the groups in terms of age, sex, weight, BMI, WC, physical activity, marital status, education level, time spent outdoors, or dietary intake at baseline.

**Table 1:** Baseline characteristics at enrollment, according to randomized assignment to intervention groups.

Characteristic^∗^	Vit D 1000 (n= 22)	Vit D 5000 (n= 22)	Vit D 1000 + NAC (n= 22)	Vit D 5000 + NAC (n= 22)	*P* value^**^
Age, year	69.5 ± 2.9	68.7 ± 4.0	70.3 ± 4.9	68.8 ± 4.6	0.561
Sex, no. (%)					
Male	9 (40.9)	10 (45.5)	14 (63.6)	7 (31.8)	0.811
Female	13 (59.1)	12 (54.5)	8 (36.4)	15 (68.2)	
Education, no. (%)					0.475
Primary school	4 (18.2)	3 (13.6)	3 (13.6)	2 (9.1)	
Secondary school	10 (45.5)	11 (50.0)	11 (50.0)	15 (68.2)	
Bachelor’s degree	8 (36.4)	8 (36.4)	6 (27.3)	3 (13.6)	
Master’s/doctoral degree	0 (0)	0 (0)	2 (9.1)	2 (9.1)	
Serum 25(OH)D, ng/mL	20.5 ± 4.0	19.3 ± 4.6	19.7 ± 4.9	18.9 ± 4.5	0.693
Time spent outdoors, min/d	61.8 ± 23.5	62.5 ± 22.2	58.7 ± 19.6	70.2 ± 19.3	0.322
BMI, kg/m^2^	27.7 ± 1.8	27.1 ± 1.7	27.9 ± 2.0	26.9 ± 1.5	0.205
Waist circumference, cm	91.7 ± 8.2	94.0 ± 9.5	93.9 ± 9.4	93.8 ± 6.3	0.783
Physical activity (RAPA score)	3.73 ± 0.985	3.86 ± 1.167	3.91 ± 0.971	3.95 ± 1.133	0.906
Dietary intakes					
Energy intake, kcal/d	2051 ± 359	1902 ± 253	1935 ± 277	1948 ± 291	0.382
Carbohydrates, g/d	280 ± 53	257 ± 44	254 ± 41	262 ± 47	0.259
Protein, g/d	70 ± 14	64 ± 10	69 ± 12	67 ± 11	0.937
Fat, g/d	79 ± 18	77 ± 15	79 ± 14	80 ± 18	0.341
PUFA, g/d	23.0 ± 7.8	22.0 ± 4.8	21.5 ± 7.0	19.8 ± 6.8	0.468
MUFA, g/d	24.0 ± 6.0	20.7 ± 5.3	23.7 ± 8.1	23.2 ± 6.8	0.368
Fiber, g/d	13.8 ± 5.2	12.8 ± 2.9	13.7 ± 4.6	14.0 ± 5.5	0.813
Zinc, mg/d	7.9 ± 2.5	8.5 ± 2.4	9.0 ± 2.4	8.2 ± 1.8	0.427
Vitamin E, mg/d	17.3 ± 8.0	16.6 ± 9.7	18.6 ± 9.7	13.8 ± 6.7	0.306
Vitamin A, μg/d	1126 ± 762	678 ± 570	710 ± 589	897 ± 825	0.130
Vitamin C, mg/d	155 ± 75	162 ± 56	154 ± 66	188 ± 79	0.332
Vitamin D, μg/d	0.056 ± 0.264	0.356 ± 0.693	0.311 ± 0.614	0.473 ± 0.611	0.140
Selenium, μg/d	0.126 ± 0.027	0.127 ± 0.028	0.128 ± 0.030	0.118 ± 0.027	0.635
Beta-carotene, μg/d	815 ± 794	417 ± 628	389 ± 595	413 ± 742	0.134

^*^Values are mean ± standard deviation unless otherwise noted.

^**^Using ANOVA or Chi-square, as appropriate.

25(OH)D, 25-hydroxyvitamin D; BMI, body mass index; MUFA, monounsaturated fatty acid; NAC, N-acetylcysteine; PUFA, polyunsaturated fatty acids.

### Biochemical parameters

**Table 2** presents the serum concentrations of Vit-D, IL-6, CRP, and NLR before and after the intervention, along with their pre- and post-treatment changes. After the 8-week intervention, the D5N group (mean: 26.0 ± 9.1 ng/ml; *p* < 0.001) and the D5 group (mean: 25.3 ± 5.2 ng/ml; *p* < 0.001) exhibited the most substantial increases in serum Vit-D compared to the D1 and D1N groups, respectively.

**Table 2:** Serum concentrations of vitamin D, immune system markers and inflammatory factors at baseline and the eighth week, according to randomized assignment to intervention groups.

	Vit D 1000 (n= 22)	Vit D 5000 (n= 22)	Vit D 1000 + NAC (n= 22)	Vit D 5000 + NAC (n= 22)	*P* value
Serum 25(OH)D, ng/mL					
Baseline	20.5 ± 4.0	19.3 ± 4.6	19.7 ± 4.9	18.9 ± 4.5	0.693^*^
Week 8	27.1 ± 4.3	44.6 ± 7.4	25.6 ± 4.3	44.9 ± 10.0	<0.001^**a,b,c,d^
Changes ^***^	6.6 ± 3.4	25.3 ± 5.2	5.9 ± 5.8	26.0 ± 9.1	<0.001^**a,b,c,d^
Serum IL-6, pg/mL					
Baseline	83.5 ± 69.1	93.3 ± 62.9	96.6 ± 85.4	81.4 ± 48.0	0.852^*^
Week 8	43.5 ± 55.1	40.7 ± 59.1	46.3 ± 104.9	27.1 ± 42.5	0.852^**^
Changes ^***^	-40.0 ± 77.6	-52.7 ± 86.0	-50.3 ± 105.7	-54.4 ± 62.2	0.852^**^
Serum CRP, mg/L					
Baseline	5.69 ± 2.10	6.26 ± 1.87	6.26 ± 1.87	6.13 ± 1.46	0.749^*^
Week 8	4.78 ± 1.46	4.45 ± 0.98	4.45 ± 0.98	4.58 ± 1.46	0.380^**^
Changes ^***^	-0.91 ± 1.61	-1.81 ± 1.72	-1.54 ± 2.15	-1.82 ± 1.58	0.380^**^
NLR					
Baseline	1.89 ± 0.77	1.73 ± 0.47	2.06 ± 0.74	1.99 ± 0.67	0.404^*^
Week 8	1.61 ± 0.67	1.42 ± 0.27	1.53 ± 0.37	1.46 ± 0.44	0.423^**^
Changes ^***^	-0.28 ± 0.76	-0.31 ± 0.32	-0.53 ± 0.60	-0.52 ± 0.51	0.423^**^
SA-β-gal activity (green area ratio%)					
Baseline	3.02 ± 1.75	3.30 ± 2.03	3.03 ± 1.98	4.20 ± 2.18	0.171^*^
Week 8	2.40 ± 1.46	1.60 ± 1.32	2.01 ± 1.33	1.68 ± 1.31	0.001^**a,b^
Changes ^***^	-0.62 ± 1.32	-1.70 ± 1.59	-1.02 ± 1.58	-2.52 ± 1.52	0.001^**a,b^

Values are mean ± standard deviation unless otherwise noted.

^*^Based on ANOVA.

^**^Based on ANCOVA adjusted for baseline values and age.

^***^Changes reflect week 8 – baseline values.

a *P*<0.05; Vit D 1000 in compare Vit D 5000.

b *P*<0.05; Vit D 1000 in compare Vit D 5000 + NAC.

c *P*<0.05; Vit D 5000 in compare Vit D 1000 + NAC.

d *P*<0.05; Vit D 1000 + NAC in compare Vit D 5000 + NAC.

25(OH)D, 25-hydroxyvitamin D; CRP, c-reactive protein; IL-6, interleukin-6; NLR, neutrophil-to-lymphocyte ratio.

No significant differences were observed between the groups after the intervention in IL-6, CRP and the NLR.

### SA-β-galactosidase staining of PBMCs

**Figure 2:**
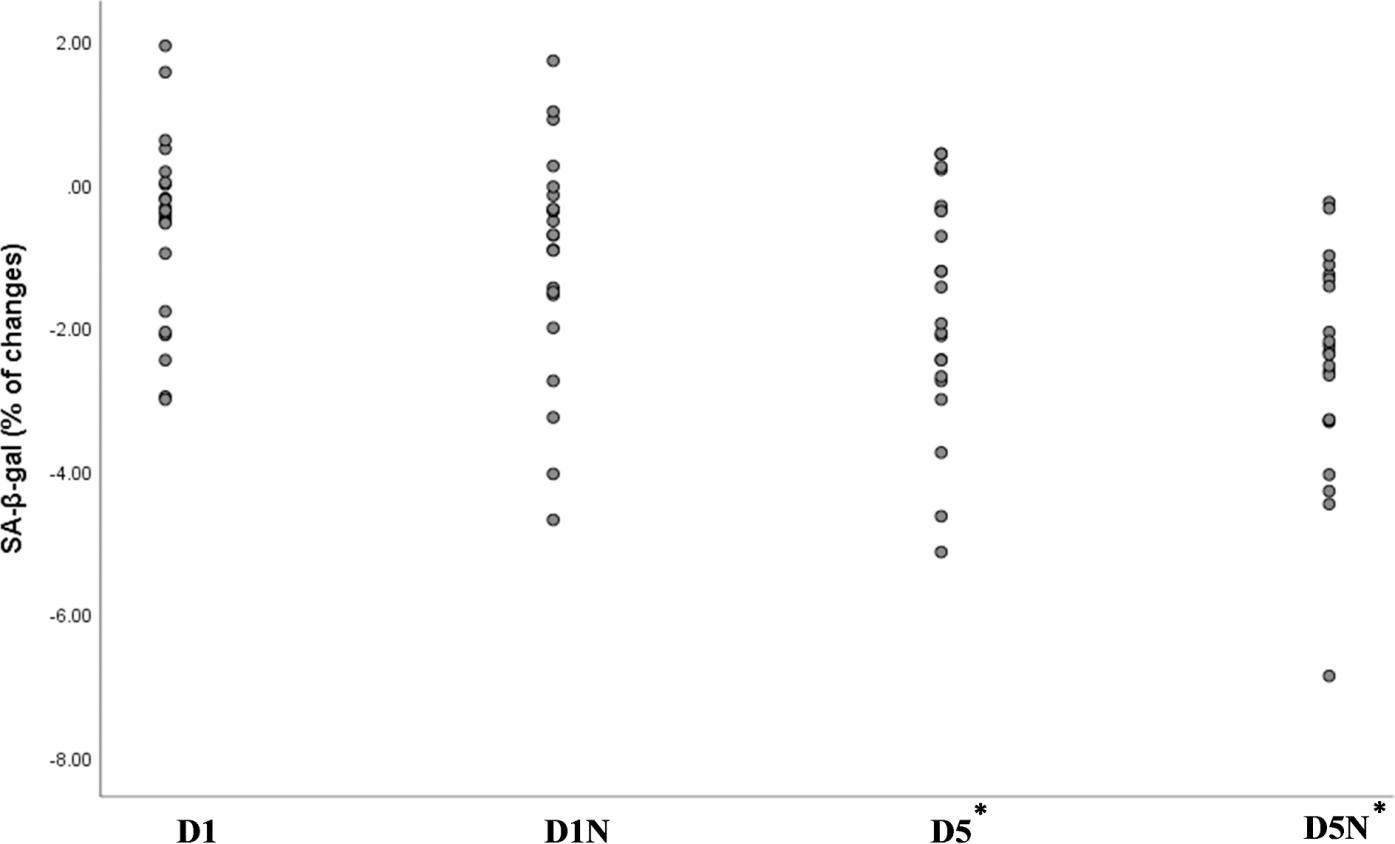
Percentage changes in the green area ratio across the four groups, representing the difference between week 8 and baseline. The green area ratio, measured using ImageJ software, quantified SA-β-gal activity. *Statistically significant p-value for the comparison of the D5 and D5N groups with the D1 group based on analysis of covariance adjusted for baseline values and age. NAC, N-acetylcysteine; D1, vitamin D 1000 IU; D5, vitamin D 5000 IU.

We developed a simple staining method to assess senescence in PBMCs, which was validated by the expression of senescence-associated genes. The results of SA-β-gal staining are presented in **Table 2** and **Figure 2**.

After the 8-week intervention, the quantification of SA-β-gal staining showed a significant decrease in the D5N (mean: −2.52% ± 1.52; *p* = 0.001) and D5 (mean: −1.70% ± 1.59; *p* = 0.001) groups compared to the D1 group. The decrease was most pronounced in the D5N group (see **Figure 2**).

### *p16*, *p21*, *IL6*, and *TNF-α* gene expression

Gene expression values were calculated as fold change. Our findings showed that D5N and D5 supplementation significantly reduced *p16* gene expression compared to the D1 group. Additionally, co-administration of NAC with D1 led to a significant decrease in *p16* gene expression compared to the D1 group.

**Figure 3:**
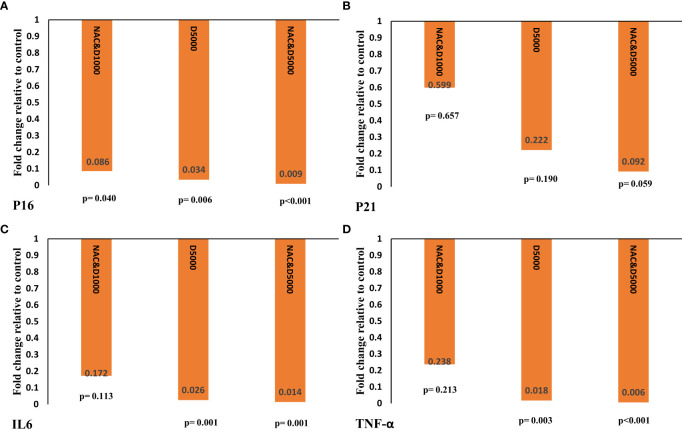
Gene expression of **(A)** *P16*, **(B)** *P21*, **(C)** *IL6*, and **(D)** *TNF-α* was calculated as fold change using the 2^−ΔΔct^ method. The ΔΔCT value was determined by subtracting the mean ΔCT changes of the intervention groups from the mean ΔCT changes of the control group. The *p*-value was obtained from an independent t-test comparing the intervention groups with the D1 control group. IL-6, interleukin-6; TNF-α, tumor necrosis factor-α; NAC: N-acetylcysteine.

The reduction in *p16* transcript levels was greatest in the D5N group (fold change: 0.009; *p* < 0.001), compared to the D5 (fold change: 0.006; *p* = 0.006) and D1N (fold change: 0.086; *p* = 0.040) groups (see **Figure 3A**).

As shown in **Figure 3B**, although *p21* gene expression decreased in the D1N, D5, and D5N groups, the reduction was not statistically significant in any group.

D5 and D5N supplementation significantly reduced *IL6* and *TNF-α* gene expression compared to the control group. However, the reduction was more pronounced in the D5N group than in the D5 group (*IL6*: fold change = 0.014, *p* = 0.001 vs. fold change = 0.026, *p* = 0.001; *TNF-α*: fold change = 0.006, *p* < 0.001 vs. fold change = 0.018, *p* = 0.003) (see **Figures 3C, D**).

### Adverse events

No adverse effects were reported in any group during the intervention.

## Discussion

Cell cycle arrest is a key characteristic of cellular senescence. The primary pathways regulating this arrest involve activation of the p53–p21 and p16 cascades, which prevent the cell from entering mitosis (30). This multi-step process leads to the overexpression of SA-β-galactosidase. β-galactosidase is a lysosomal hydrolase associated with replicative aging and the presence of autophagic vacuoles in senescent cells. Therefore, the SA-β-gal assay is the simplest and most commonly used method to evaluate the impact of various conditions or substances on senescent cell formation (31). In this study, we investigated the effects of NAC supplementation and two different doses of Vit-D on p16 and p21 gene expression, as well as SA-β-gal activity. We aimed to identify a safe and effective supplementation strategy to mitigate immunosenescence in older adults.

Accumulation of senescent cells in tissues and organs and subsequent age-related dysfunction mostly occur in older adults due to an impaired immune response that hinders the clearance of these cells (14, 32).

Our results showed that Vit-D supplementation at a dose of 5000 IU, alone or in combination with NAC, significantly downregulated *p16*, *IL6*, and *TNF-α* expression, along with reducing SA-β-gal activity in PBMCs, in Vit-D–deficient older individuals compared to the D1 group. However, these changes were higher in the D5N than in the D5 group. Additionally, our data showed that co-administration of NAC with 1000 IU of Vit-D significantly downregulated *p16* transcript levels in PBMCs compared to Vit-D (1000 IU) alone.

Age-related oxidative stress and redox imbalance induce cellular senescence and SASP secretion (33). Meanwhile, two key factors contributing to immune cell dysfunction with aging are a significant decline in leukocyte and plasma glutathione (GSH) levels and Vit-D deficiency (34). Consistent with these findings, our results showed that NAC (a GSH precursor) and Vit-D supplementation reduced senescence markers in older individuals.

Several studies have identified a negative correlation between Vit-D levels and senescence markers, such as *p16* expression and SASP production. Chen et al. reported that Vit-D supplementation inhibited cellular senescence and SASP secretion by reducing oxidative stress and DNA damage, thereby inactivating the *p21* and *p16* signaling pathways in Vit-D–deficient mice (35). In another study, Yang et al. reported that Vit-D supplementation inhibited osteocyte senescence and SASP secretion induced by natural aging by downregulating the *p16* transcript (36). Furthermore, consistent with our findings on the enhanced effects of Vit-D with NAC supplementation, Chen et al. reported that NAC significantly reduced cellular senescence induced by Vit-D deficiency in mice. In other words, NAC was found to be as effective as Vit-D supplementation (17). Another study by these authors reported that NAC supplementation inhibited SASP production, including IL-6 and TNF-α, and reduced SA-β-gal activity in mice (24). Our previous clinical trial on individuals with obesity demonstrated that NAC supplementation significantly reduced serum IL-6 and CRP levels while downregulating *p16* and *IL6* gene expression in adipose tissue (15). Similar to aging, obesity is associated with the accumulation of senescent cells and accelerates age-related diseases. Therefore, targeting senescent cells may help prevent premature biological aging. Furthermore, a study in older women reported that NAC improves immune function by increasing the leukocyte glutathione pool and reducing plasma and leukocyte inflammatory markers, such as TNF-α (34). An *in-vitro* study demonstrated that NAC suppressed the secretion and gene expression of *TNF-α* and *IL-6* in lipopolysaccharide-activated macrophages, thereby modulating the inflammatory response (37).

Based on our findings and similar studies, NAC supplementation appears to inhibit SASP secretion both directly and indirectly. Directly, it reduces ROS through its thiol group, while indirectly, it enhances the cellular antioxidant pool as a GSH precursor by stimulating cytosolic enzymes involved in GSH recovery. Additionally, NAC suppresses pro-inflammatory gene expression by blocking the translocation and nuclear activation of the NF-κB transcription factor (38).

On the other hand, Vit-D exerts its effects through genomic pathways by modulating proliferation, differentiation, and cytokine production in various immune cells that express Vit-D receptors (VDRs) and metabolizing enzymes (39). Beyond the genomic pathway, Vit-D also has non-canonical effects on aging and age-related diseases. Although it does not possess free radical scavenging activity, Vit-D exerts antioxidant and anti-inflammatory effects by regulating the expression of anti-aging genes such as *Nrf2* and *Klotho*. This, in turn, upregulates detoxification enzymes, including glutathione peroxidase/reductase and superoxide dismutase (40). Vit-D can also function as a potent indirect antioxidant and modulate SASP secretion by inhibiting cyclooxygenase-2, maintaining genomic stability, downregulating *TNF-α* gene expression, and regulating key metabolic pathways in senescent cells. Notably, it inhibits NF-κB translocation, thereby controlling inflammation (22, 41).

We hypothesized that NAC combined with Vit-D would further reduce oxidative stress and enhance immunity by decreasing SA-β-gal activity and senescence-related gene expression in the PBMCs of older adults. Our findings support this hypothesis, as the greatest reduction in *p16*, *p21*, *IL6*, and *TNF-α* gene expression was observed in the D5N group. Notably, co-administration of NAC with Vit-D significantly reduced *p16* gene expression in the D1N group compared to the D1 group. Consistent with our findings, several studies have reported that Vit-D supplementation combined with NAC enhances their effectiveness in reducing oxidative stress and inflammation. In a study on neonates with hypoxic-ischemic encephalopathy, Jenkins et al. reported that co-administration of NAC with Vit-D significantly reduced oxidative stress in both plasma and the CNS while improving CNS energetics (42). In another study by these authors, co-administration of Vit-D with NAC reduced neuroinflammation in neonatal male rats (43).

Recent reports have shown a positive association between GSH and Vit-D concentrations in adults (31). To explain the additive effects of taking Vit-D and NAC together, Vit-D induces glutathione reductase synthesis, enhancing GSH biosynthesis (44). Conversely, GSH deficiency and increased oxidative stress reduce the expression of genes involved in Vit-D metabolism, impairing its bioavailability (45). Therefore, co-administering NAC—an absorbable and stable GSH precursor—with Vit-D may enhance therapeutic efficacy by simultaneously modulating GSH biosynthesis and Vit-D metabolism.

Recently, NLR has been introduced as a novel hematological marker of systemic inflammation (46). Our findings showed that the standard dose of Vit-D alone did not significantly alter NLR in Vit-D–deficient older adults. However, NLR was significantly reduced from baseline in the D1N, D5, and D5N groups. These results are consistent with the effects of these supplements on *p16* gene expression. NLR is a simple, inexpensive, and widely available marker that provides insight into a person’s immune status (46). The accumulation of senescent cells and the subsequent inflammation caused by SASP products can lead to an increased NLR (47, 48). A recent study in a healthy population reported that NLR was positively associated with age and BMI (49). These findings are consistent with the fact that the expression of senescence-related genes increases with age and BMI (50). In addition, evidence suggests that age-related diseases in which cellular senescence plays a role in their pathogenesis—such as diabetes (51, 52), cardiovascular diseases (51, 52), and neurodegenerative diseases (53, 54)—are associated with a high NLR. In light of these findings, our results suggest that an increase in NLR, alongside other senescence markers, may be a hallmark of immunosenescence.

Consistent with our findings, some studies on COVID-19 patients have reported that NAC and Vit-D supplementation can significantly reduce NLR by controlling oxidative stress and cytokine release (55, 56).

Based on the results of the current study and supporting evidence, NAC and Vit-D supplements may be considered senotherapeutic agents. When used together, they could yield enhanced effects.

This study had certain limitations. Since cholecalciferol is the primary treatment for Vit-D deficiency, providing it to all participants was an ethical necessity. Consequently, the control group received a maintenance dose of 1000 IU of Vit-D, meaning the study lacked a true placebo group. Additionally, we were unable to assess the independent effects of NAC in Vit-D–deficient older adults.

Based on our review of the scientific literature, this study is the first to investigate SA-β-gal staining in PBMCs as a simple and reliable marker of cellular senescence. Another strength of this study is the evaluation of two different doses of Vit-D, the co-administration of NAC with Vit-D, and their potential synergistic effects.

## Conclusion

In conclusion, this randomized, double-blind clinical trial demonstrated that a loading dose of Vit-D significantly reduces SA-β-gal activity and the expression of *p16*, *IL-6*, and *TNF-α* genes in the PBMCs of Vit-D–deficient older adults compared to the standard dose. However, co-administration of NAC with both standard and loading doses of Vit-D enhanced these senotherapeutic effects. Our findings support a safe supplementation strategy to reduce senescence in immune cells, utilizing a simple and reliable method for PBMC senescence analysis.

## Data Availability

The datasets presented in this study can be found in online repositories. The names of the repository/repositories and accession number(s) can be found in the article/**Supplementary Material**.
