# Corrigendum: Co-administration of vitamin D and N-acetylcysteine to modulate immunosenescence in older adults with vitamin D deficiency: a randomized clinical trial

**DOI:** 10.3389/fimmu.2025.1627178

**Published:** 2025-05-29

**Authors:** Samira Rastgoo, Katayoun Pourvali, Seyed Ahmad Raeissadat, Ghazaleh Eslamian, Hamid Zand

**Affiliations:** ^1^ Department of Cellular and Molecular Nutrition, Faculty of Nutrition and Food Technology, National Nutrition and Food Technology Research Institute, Shahid Beheshti University of Medical Sciences, Tehran, Iran; ^2^ Physical Medicine and Rehabilitation Department, Shahid Modarres Hospital, Shahid Beheshti University of Medical Sciences, Tehran, Iran

**Keywords:** immune function, geroscience, cellular senescence, nutrition, aging

In the published article, there was a typographical error in the legend of **Table 2**. “Vit D 5000” and “Vit D 5000 + NAC” were mistakenly written as “Vit D 50000” and “Vit D 50000 + NAC”. The correct terms are “Vit D 5000” and “Vit D 5000 + NAC”. The corrected legend appears below.

“^a^P<0.05; Vit D 1000 in compare Vit D 5000

^b^P<0.05; Vit D 1000 in compare Vit D 5000 + NAC

^c^P<0.05; Vit D 5000 in compare Vit D 1000 + NAC

^d^P<0.05; Vit D 1000 + NAC in compare Vit D 5000 + NAC”

In the published article, there was a typographical error in the first row of **Tables 1** and **2**. “Vit D 5000” and “Vit D 5000 + NAC” were mistakenly written as “Vit D 50000” and “Vit D 50000 + NAC”. The correct terms are “Vit D 5000” and “Vit D 5000 + NAC”. The corrected versions of **Table 1** and **Table 2**, along with their captions “Table 1. Baseline characteristics at enrollment, according to randomized assignment to intervention groups.

**Table 1:** Baseline characteristics at enrollment, according to randomized assignment to intervention groups.

Characteristic^∗^	Vit D 1000 (n= 22)	Vit D 5000 (n= 22)	Vit D 1000 + NAC (n= 22)	Vit D 5000 + NAC (n= 22)	*P* value^**^
Age, year	69.5 ± 2.9	68.7 ± 4.0	70.3 ± 4.9	68.8 ± 4.6	0.561
Sex, no. (%)					
Male	9 (40.9)	10 (45.5)	14 (63.6)	7 (31.8)	0.811
Female	13 (59.1)	12 (54.5)	8 (36.4)	15 (68.2)	
Education, no. (%)					0.475
Primary school	4 (18.2)	3 (13.6)	3 (13.6)	2 (9.1)	
Secondary school	10 (45.5)	11 (50.0)	11 (50.0)	15 (68.2)	
Bachelor’s degree	8 (36.4)	8 (36.4)	6 (27.3)	3 (13.6)	
Master’s/doctoral degree	0 (0)	0 (0)	2 (9.1)	2 (9.1)	
Serum 25(OH)D, ng/mL	20.5 ± 4.0	19.3 ± 4.6	19.7 ± 4.9	18.9 ± 4.5	0.693
Time spent outdoors, min/d	61.8 ± 23.5	62.5 ± 22.2	58.7 ± 19.6	70.2 ± 19.3	0.322
BMI, kg/m^2^	27.7 ± 1.8	27.1 ± 1.7	27.9 ± 2.0	26.9 ± 1.5	0.205
Waist circumference, cm	91.7 ± 8.2	94.0 ± 9.5	93.9 ± 9.4	93.8 ± 6.3	0.783
Physical activity (RAPA score)	3.73 ± 0.985	3.86 ± 1.167	3.91 ± 0.971	3.95 ± 1.133	0.906
Dietary intakes					
Energy intake, kcal/d	2051 ± 359	1902 ± 253	1935 ± 277	1948 ± 291	0.382
Carbohydrates, g/d	280 ± 53	257 ± 44	254 ± 41	262 ± 47	0.259
Protein, g/d	70 ± 14	64 ± 10	69 ± 12	67 ± 11	0.937
Fat, g/d	79 ± 18	77 ± 15	79 ± 14	80 ± 18	0.341
PUFA, g/d	23.0 ± 7.8	22.0 ± 4.8	21.5 ± 7.0	19.8 ± 6.8	0.468
MUFA, g/d	24.0 ± 6.0	20.7 ± 5.3	23.7 ± 8.1	23.2 ± 6.8	0.368
Fiber, g/d	13.8 ± 5.2	12.8 ± 2.9	13.7 ± 4.6	14.0 ± 5.5	0.813
Zinc, mg/d	7.9 ± 2.5	8.5 ± 2.4	9.0 ± 2.4	8.2 ± 1.8	0.427
Vitamin E, mg/d	17.3 ± 8.0	16.6 ± 9.7	18.6 ± 9.7	13.8 ± 6.7	0.306
Vitamin A, μg/d	1126 ± 762	678 ± 570	710 ± 589	897 ± 825	0.130
Vitamin C, mg/d	155 ± 75	162 ± 56	154 ± 66	188 ± 79	0.332
Vitamin D, μg/d	0.056 ± 0.264	0.356 ± 0.693	0.311 ± 0.614	0.473 ± 0.611	0.140
Selenium, μg/d	0.126 ± 0.027	0.127 ± 0.028	0.128 ± 0.030	0.118 ± 0.027	0.635
Beta-carotene, μg/d	815 ± 794	417 ± 628	389 ± 595	413 ± 742	0.134

^*^Values are mean ± standard deviation unless otherwise noted.

^**^Using ANOVA or Chi-square, as appropriate.

25(OH)D, 25-hydroxyvitamin D; BMI, body mass index; MUFA, monounsaturated fatty acid; NAC, N-acetylcysteine; PUFA, polyunsaturated fatty acids.

**Table 2:** Serum concentrations of vitamin D, immune system markers and inflammatory factors at baseline and the eighth week, according to randomized assignment to intervention groups.

	Vit D 1000 (n= 22)	Vit D 5000 (n= 22)	Vit D 1000 + NAC (n= 22)	Vit D 5000 + NAC (n= 22)	*P* value
Serum 25(OH)D, ng/mL					
Baseline	20.5 ± 4.0	19.3 ± 4.6	19.7 ± 4.9	18.9 ± 4.5	0.693^*^
Week 8	27.1 ± 4.3	44.6 ± 7.4	25.6 ± 4.3	44.9 ± 10.0	<0.001^**a,b,c,d^
Changes ^***^	6.6 ± 3.4	25.3 ± 5.2	5.9 ± 5.8	26.0 ± 9.1	<0.001^**a,b,c,d^
Serum IL-6, pg/mL					
Baseline	83.5 ± 69.1	93.3 ± 62.9	96.6 ± 85.4	81.4 ± 48.0	0.852^*^
Week 8	43.5 ± 55.1	40.7 ± 59.1	46.3 ± 104.9	27.1 ± 42.5	0.852^**^
Changes ^***^	-40.0 ± 77.6	-52.7 ± 86.0	-50.3 ± 105.7	-54.4 ± 62.2	0.852^**^
Serum CRP, mg/L					
Baseline	5.69 ± 2.10	6.26 ± 1.87	6.26 ± 1.87	6.13 ± 1.46	0.749^*^
Week 8	4.78 ± 1.46	4.45 ± 0.98	4.45 ± 0.98	4.58 ± 1.46	0.380^**^
Changes ^***^	-0.91 ± 1.61	-1.81 ± 1.72	-1.54 ± 2.15	-1.82 ± 1.58	0.380^**^
NLR					
Baseline	1.89 ± 0.77	1.73 ± 0.47	2.06 ± 0.74	1.99 ± 0.67	0.404^*^
Week 8	1.61 ± 0.67	1.42 ± 0.27	1.53 ± 0.37	1.46 ± 0.44	0.423^**^
Changes ^***^	-0.28 ± 0.76	-0.31 ± 0.32	-0.53 ± 0.60	-0.52 ± 0.51	0.423^**^
SA-β-gal activity (green area ratio%)					
Baseline	3.02 ± 1.75	3.30 ± 2.03	3.03 ± 1.98	4.20 ± 2.18	0.171^*^
Week 8	2.40 ± 1.46	1.60 ± 1.32	2.01 ± 1.33	1.68 ± 1.31	0.001^**a,b^
Changes ^***^	-0.62 ± 1.32	-1.70 ± 1.59	-1.02 ± 1.58	-2.52 ± 1.52	0.001^**a,b^

Values are mean ± standard deviation unless otherwise noted.

^*^Based on ANOVA.

^**^Based on ANCOVA adjusted for baseline values and age.

^***^Changes reflect week 8 – baseline values.

a *P*<0.05; Vit D 1000 in compare Vit D 5000.

b *P*<0.05; Vit D 1000 in compare Vit D 5000 + NAC.

c *P*<0.05; Vit D 5000 in compare Vit D 1000 + NAC.

d *P*<0.05; Vit D 1000 + NAC in compare Vit D 5000 + NAC.

25(OH)D, 25-hydroxyvitamin D; CRP, c-reactive protein; IL-6, interleukin-6; NLR, neutrophil-to-lymphocyte ratio.

Table 2. Serum concentrations of Vitamin D, immune system markers and inflammatory factors at baseline and the eighth week, according to randomized assignment to intervention groups” appear below.

The authors apologize for this error and state that this does not change the scientific conclusions of the article in any way. The original article has been updated.

