# Supplementary material for: Co-administration of vitamin D and N-acetylcysteine to modulate immunosenescence in older adults with vitamin D deficiency: a randomized clinical trial
Source: Front Immunol. 2025 May 12;16:1570441. doi: 10.3389/fimmu.2025.1570441 (PMC12104082; doi:10.3389/fimmu.2025.1570441)
Supplement: Supplementary file 1 [file DataSheet1.pdf]

Appendix

Shahid Beheshti University of Medical Sciences and Health Services

Faculty of Nutritional Sciences and Food Technology

Participant demographic and anthropometric information

Research Project: *Comparison of the Effects of Vitamin D and N-Acetylcysteine Supplementation on the Expression of Cellular Senescence Genes and Inflammatory Factors in Older Adults with Vitamin D Deficiency: A Double-Blind Randomized Clinical Trial*

Participant Code……

A. General Information

Age: ....; Gender....................; Education: ....; Employment Status: ....;

Marital Status: …………….

B. Anthropometric data

Weight: ………. kg;

Height: …… cm;

Waist circumference: …………cm;

Body Mass Index: ………. Kg/m^2

C: Serum vitamin D: ………. ng/ml

D. Exposure to sunlight

- How much time do you spend in sunlight each day, approximately?

No exposure to sunlight ◯ 10 minutes to an hour ◯ 1-2 hours ◯ More than 2 hours ◯

- What time of day do you typically expose yourself to sunlight? *(Please specify the time range)*

7-10 ◯ 10-15 ◯ 15-17 ◯

- What parts of your body are usually exposed to sunlight without covering? *(Check all that apply)*

"Face" ◯ "Hands from wrist to fingertips" ◯

"Hands from the arms to fingertips" ◯ "Legs" ◯

Other: ……………

- Do you use sunscreen when exposed to sunlight? *(Yes/No)*

E. Medical Conditions

(Please indicate if you suffer from any of the following conditions)

- Chronic and acute inflammatory diseases ; …………………..
- Infectious diseases; ……………..
- Diabetes and thyroid disorders; …………………
- Alzheimer's disease or dementia; ……………..
- Hypercalcemia ◯ Electrolyte imbalance ◯

- Renal dysfunction ◯ Hepatobiliary dysfunction ◯
- Gastric hemorrhage ◯ Arrhythmia ◯ Asthma ◯

F. Medications (Use of Drugs)

(Please indicate if you are currently taking any of the following medications)

Anticonvulsants ◯ Anti-tuberculosis drugs ◯ Glucocorticoids ◯ HIV drugs ◯

Hydrochlorothiazide ◯ Magnesium oxide ◯

Have you taken vitamin D, N-acetylcysteine, or antioxidant supplements in the last three months? ◯

Please list any other medications you are currently taking:………………………………………

G. Alcohol and smoking

Alcohol intake ◯ Smoking ◯
