# Supplementary material for: Co-administration of vitamin D and N-acetylcysteine to modulate immunosenescence in older adults with vitamin D deficiency: a randomized clinical trial
Source: Front Immunol. 2025 May 12;16:1570441. doi: 10.3389/fimmu.2025.1570441 (PMC12104082; doi:10.3389/fimmu.2025.1570441)
Supplement: Supplementary file 2 [file DataSheet2.pdf]

Protocol for Preparing Staining Solution to Detect Senescence-associated Beta-Galactosidase Activity (1)

Required Solutions

1. **Fixative Solution**

Materials and Preparation Method:

Row	Substance Name	Required Volume	Required Solvent Volume (PBS)	Solution Concentration (Volume Percent)	Solvent	Final solution volume	Warning
1	Formaldehyde 37%	1ml	48.9 ml	2%	PBS	50 ml	Toxic Substance
2	Glutaraldehyde 50%	100 microliters		0.2%	PBS		Toxic Substance

It can be stored for one month at room temperature.

Measure the specified amounts of the two substances and dilute with PBS to the required volume.

2. **Citric Acid Solution**

Materials and Preparation Method:

Row	Substance Name	Required Weight (g)	Solvent	Solution Concentration	Final solution volume	Molecular Weight (g/mol)	Warning
1	Citric Acid C6H8O7.H2O	0.210 g	Distilled Water	100 mM	10 ml	210.14	Skin Irritant

It can be stored for several weeks at room temperature.

Weigh the specified amount of the substance, dissolve it in distilled water and adjust to the required volume.

3. **Disodium Phosphate Solution**

Materials and Preparation Method:

Row	Substance Name	Required Weight (g)	Solvent	Solution Concentration	Final solution volume	Molecular Weight (g/mol)	Warning
1	NaH2PO4.H2O	0.275 g	Distilled Water	200 mM	10 ml	137.99	Skin Irritant

Alternatively, the following combination can be used:

Row	Substance Name	Required Weight (g)	Solvent	Solution Concentration	Final solution volume	Molecular Weight (g/mol)	Warning
1	$Na_2HPO_4.2H_2O$	0.355 g	Distilled Water	200 mM	10 ml	177.99	Skin Irritant

It can be stored for several weeks at room temperature.

Weigh the specified amount of the substance, dissolve it in distilled water and adjust to the required volume.

4. Citric Acid Solution in Sodium Phosphate Buffer (0.2 M)

Materials and Preparation Method:

Row	Substance Name	Required Volume (ml)	Final solution volume	pH	Warning
1	Citric Acid Solution 100 mM (Solution #2)	36.85 ml	100 ml	6	Skin Irritant
2	Sodium Phosphate Solution (Solution #3)	63.15 ml			

It can be stored for one month at room temperature.

Combine the specified volumes of the two solutions and adjust the pH to 6.

5. X-gal Solution

Materials and Preparation Method:

Row	Substance Name	Required Weight (g)	Solution Concentration	Final solution volume	Warning
1	X-gal	0.02 g	20 mg/ml	1 ml	
2	Dimethylformamide N, N-dimethylformamide	-	1 ml		Toxic Substance

It is recommended to prepare it freshly, but it can be stored for two weeks at -20 ° C.
In this study, *X-gal* (BioBasic, Canada) was used

Weigh the specified amount of X-gal, dissolve it in dimethylformamide, and adjust to the required volume.

6. **Potassium Hexacyanoferrate II Trihydrate Solution**

Materials and Preparation Method:

Row	Substance Name	Required Weight (g)	Solvent	Solution Concentration	Final solution volume	Molecular Weight (g/mol)	Warning
1	Hexacyanoferrate Trihydrate K4[Fe (CN)6] 3H2O	0.042 g	Distilled Water	100 mM	1 ml	422.39	Hazardous Substance

It can be stored for several months at 4°C in a refrigerator.

Weigh the specified amount of the substance, dissolve it in water, and adjust to the required volume.

7. **Potassium Hexacyanoferrate III Solution**

Materials and Preparation Method:

Row	Substance Name	Required Weight (g)	Solvent	Solution Concentration	Final solution volume	Molecular Weight (g/mol)	Warning
1	Hexacyanoferrate K3[Fe(CN)6]	0.032 g	Distilled Water	100 mM	1 ml	329.24	Hazardous Substance

It can be stored for several months at 4°C in a refrigerator.

Weigh the specified amount of the substance, dissolve it in water, and adjust to the required volume.

8. **Sodium Chloride (NaCl) Solution**

Materials and Preparation Method:

Row	Substance Name	Required Weight (g)	Solvent	Solution Concentration	Final solution volume	Molecular Weight (g/mol)	Warning
1	Sodium Chloride NaCl	29.22 g	Distilled Water	5 M	100 ml	58.44	-

It can be stored for one month at room temperature.

Weigh the specified amount of sodium chloride, dissolve it in water, and adjust to the required volume.

9. **Magnesium Chloride Hexahydrate Solution**

Materials and Preparation Method:

Row	Substance Name	Required Weight (g)	Solvent	Solution Concentration	Final solution volume	Molecular Weight (g/mol)	Warning
1	Magnesium Chloride Hexahydrate MgCl2.6H2O	20.33 g	Distilled Water	1 M	100 ml	203.30	-

It can be stored for one month at room temperature.

Weigh the specified amount of magnesium chloride, dissolve it in water, and adjust to the required volume.

10. **Staining Solution**

Materials and Preparation Method:

Row	Substance Name	Required Volume (µl)	Solution Concentration	Final solution volume
1	Citric Acid Solution in Sodium Phosphate Buffer (Solution #4)	200 µl	40 mM	1 ml
2	Potassium Hexacyanoferrate II Trihydrate Solution (Solution #6)	50 µl	5 mM	
3	Potassium Hexacyanoferrate III Solution (Solution #7)	50 µl	5 mM	
4	Sodium Chloride Solution (Solution #8)	30 µl	150 mM	
5	Magnesium Chloride Hexahydrate Solution (Solution #9)	2 µl	2 mM	
6	X-gal Solution (Solution #5)	50 µl	1 mg/ ml	

The solution should be freshly prepared and used immediately.

Measure the specified amounts of each solution, combine them, and dilute to the required volume with distilled water.

Reference:

1. Debacq-Chainiaux F, Erusalimsky JD, Campisi J, Toussaint O. Protocols to detect senescence-associated beta-galactosidase (SA-betagal) activity, a biomarker of senescent cells in culture and in vivo. Nat Protoc. 2009;4(12):1798-806.
